# De novo sequencing and transcriptome assembly of *Arisaema heterophyllum* Blume and identification of genes involved in isoflavonoid biosynthesis

**DOI:** 10.1038/s41598-018-35664-1

**Published:** 2018-12-05

**Authors:** Chenkai Wang, Jinhang Zhu, Miaomiao Liu, Qingshan Yang, Jiawen Wu, Zegeng Li

**Affiliations:** 1 Anhui University of Chinese Medicine and Anhui Academy of Chinese Medicine, Hefei, 230038 China; 2 Anhui Medical University, Hefei, 230032 China; 3 Key Laboratory of Xin’an Medicine, Ministry of Education, Anhui University of Chinese Medicine, Hefei, 230038 China; 4 Synergetic Innovation Center of Anhui Authentic Chinese Medicine Quality Improvement, Hefei, 230012 China; 5 The First Affiliated Hospital of Anhui University of traditional Chinese Medicine, Anhui, 230038 China; 6 Key Laboratory of Respiratory Diseases, State Administration of Traditional Chinese Medicine of the People’s Republic of China, Anhui, 230038 China

## Abstract

*Arisaema heterophyllum* Blume (*AhBl*) is one of the valued medicinal plants. However, its genetic information is limited, which impedes further studies of this valuable resource. To investigate the genes involved in the isoflavonoid biosynthesis, we deeply performed transcriptome sequencing for *AhBl*. An average of 10.98 Gb clean reads were obtained based on root, tuber and leaf tissues, and 109,937 unigenes were yielded after de novo assembly. In total, 72,287 of those unigenes were annotated in at least one public database. The numbers of expressed unigenes in each tissue were 35,686, 43,363 and 47,783, respectively. The overall expression levels of transcripts in leaf were higher than those in root and tuber. Differentially expressed genes analysis indicated that a total of 12,448 shared unigenes were detected in all three tissues, 10,215 of which were higher expressed in tuber than that in root and leaf. Besides, 87 candidate unigenes that encode for enzymes involved in biosynthesis of isoflavonoid were identified and analyzed, and some key enzyme genes were experimentally validated by quantitative Real-Time PCR (qRT-PCR). This study provides a unique dataset for the systematic analysis of *AhBl* functional genes and expression characteristics, and facilitates the future study of the pharmacological mechanism of *AhBl*.

## Introduction

*Arisaema heterophyllum* Blume (*AhBl*) is a perennial medicinal plant of the *Araceae* family. The dried tuber of *AhBl*, called Arisaema, is a traditional Chinese medicine with a long history usage. Approximately 150 species of Arisaema are distributed around the world^1^, and most of these species are found in Yunnan Province, China^2^. *AhBl* has been reported to possess different pharmacological activities, mainly including anti-tumor^3–5^, antibacterial^6^, anticonvulsant^7^, analgesic^8,9^ and anti-inflammatory^10^. In addition, it has a more complex chemical composition and has been detected the presence of alkaloids, flavonoids, plant lectins, lignans and terpenes^11,12^. Flavonoids are widely distributed in the plant kingdom and their polyphenolic compounds play important roles in regulating the activities of enzymes. The flavonoids are also significant chemical components of *AhBl*, which content relating to the different growth stages of *AhBl*^13^. The total flavonoids content can be used for quantitative evaluation of *AhBl*^14^. Isoflavonoid is a crucial subgroup of flavonoids with anti-cancer, promoting growth, antioxidant and enhancing immunity^15^. Despite the fact of its important medicinal value, there is limited information available for the biosynthesis of isoflavonoid.

Presently, the study of *AhBl* mainly focused on the extraction, identification and pharmacology of active ingredients. While the information on the functions of its genes is still very scarce, which limits the further development and use of this medicinal herb. In recent years, RNA sequencing technology (RNA-seq) has changed the research model of traditional molecular biology, and large-scale transcripts can be obtained more effectively and efficiently. RNA-seq is a particularly efficacious way to decipher novel gene functions, and provide information on gene expression and regulation^16,17^, especially in plants without reference genome. RNA-seq has been extensively applied to identify the functional genes of herbal medicine^18,19^. Large numbers of Chinese herbal medicines have been performed de novo sequencing and analysis of their transcriptome data. For instance, many candidate genes involved in biosynthesis of environmental stress-associated pathways were identifed in *Panax ginseng*^20^, most known genes participated in biosynthesis of benzoic acid were also identified in the transcriptome of *Pinellia ternata*^21^, the gene expression indices were analyzed in the unigene dataset of *Azadirachta indica*^22^, and 70% of the ESTs were generated in the transcriptome study of *Maize*^23^.

Herein, we obtained the transcriptome data from three tissues of *AhBl* by RNA-seq. In total, 109,937 unigenes were assembled to construct *AhBl* transcriptome. Functional annotation and analysis on the levels of gene expression were performed for all-unigenes. Genes encoding some key enzymes involved in isoflavonoid biosynthesis pathway were identified. The transcriptome data provides a valuable resource for improving the yield of isoflavonoid through metabolic engineering and lays the foundation for future studies of functional genes from *AhBl*.

## Results

### RNA-seq and Transcriptome De novo Assembly

*AhBl* cDNA libraries derived from three different tissues, namely root, tuber and leaf, which were individually used for sequencing, assembly and analysis. Illumina HiSeq 4,000 sequencing generated 248.55 Mb raw paired-end reads. After removing low quality reads, ambiguous reads and adaptor sequences, a total of 32.93 Gb clean reads were obtained with the average Q20 of 95.89% (sequencing error rate <1%) (Supplementary Table S1). Then the clean reads of three tissues were assembled into 255,486 transcripts by the Trinity software. The total number of transcripts per tissue was 64,434, 89,452 and 101,600, respectively (Supplementary Table S2). To acquire an overview of the transcriptome of *AhBl*, the assembled transcripts of these three tissues were used to cluster into 51,310, 67,957 and 80,957 unigenes, respectively. By using the TGI Clustering (TGICL) software, unigenes of the three tissues were then clustered to all-unigenes (109,937) with a median length of 1,194 bp, an N50 length of 1,988 bp and a GC percentage of 46.81 (Supplementary Table S3). Moreover, the length distributions of all-unigenes revealed that 67,954 (61.81%) unigenes were more than 500 bp, 41,111 (37.40%) were more than 1,000 bp, and 23,461 (21.34%) were more than 1,500 bp (Supplementary Fig. S1). These results demonstrated that the integrity of all the unigenes were good enough for downstream analysis.

### Numbers of Expressed Transcripts across Root, Tuber and Leaf Tissues

**Figure 1:**
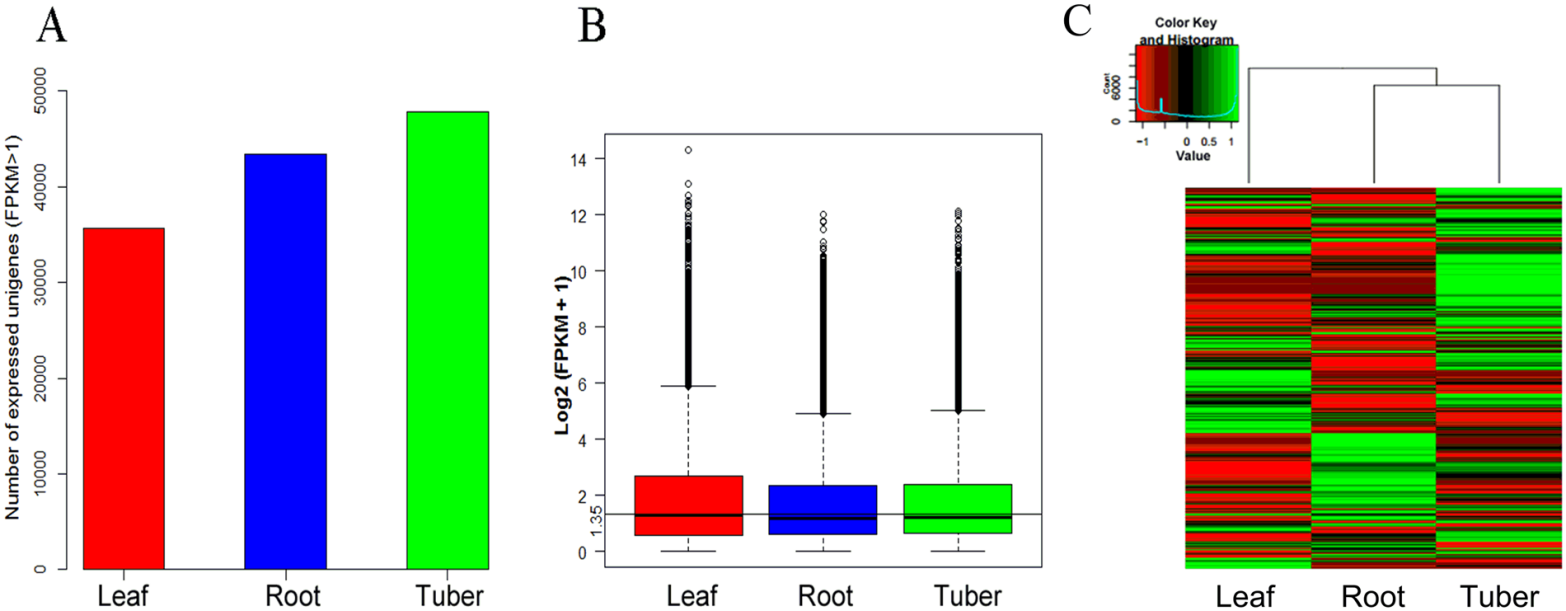
Overview of unigenes expression profiles and heatmap in the three tissues of *AhBl*. (**A**) Numbers of expressed unigenes (FPKM > 1) in three tissues. (**B**) Boxplot of unigenes expressed in three tissues. The X axis represents the samples. The Y axis represents the log2 (FPKM + 1) values. (**C**) Heatmap of unigenes expressed in the three tissues. The intensity of the colour scheme is scaled to the log2 (FPKM + 1) expression values and green and red represent high and low expression levels, respectively.

To detect the expressed transcripts, all of the expressed unigenes in each tissue were calculated based on FPKM > 1. The number of expressed unigenes across the three tissues was 35,686, 43,363 and 47,783, respectively (Fig. 1A). In addition, the FPKM data was screened through using a log2 transformation that added one to all FPKM values to avoid log2 (0) meaningless. We observed that the overall expression levels of transcripts in leaf was relatively higher than in root and tuber (Fig. 1B). Low expression unigenes were filtered according to geometric mean of (FPKM + 1)<3 as the threshold. 86,561 unigenes were used for hierarchical clustering analysis. It showed that root and tuber were in the same branch, illustrating the overall expression levels of transcripts in the two tissues clustered more similar (Fig. 1C), which is consistent with the plants growth condition, leaves grow above the ground, and roots and tubers grow underneath the ground (Supplementary Fig. S2).

### Unigenes Functional Annotation

To achieve more information and complete functional annotation, all assembled unigene sequences were searched against various databases, including Non-Redundant (NR), Nucleotide (NT), SwissProt, Kyoto Encyclopedia of Genes and Genomes (KEGG), Interpro, Cluster of Orthologous Groups of Proteins (COG) and Gene Ontology (GO) using BLASTx program (E-value ≤ 1e-5). According to the analysis of venn diagram (Supplementary Fig. S3A), 24,614 unigenes were co-annotated by the five databases. All functional annotations were outlined in Table 1. Among 72,287 annotated unigenes, 67,065 (61.00%) of the annotated unigenes were aligned to NR database and 51,489 (46.84%) were annotated in the NT database. 47,752 unigenes (43.44%) were annotated in the SwissProt and 53,451 unigenes (48.62%) were matched to the KEGG database. The number of all-unigenes annotated to the COG, Interpro and GO databases was 34,918 (31.76%), 49,617 (45.13%) and 8,496 (7.73%), respectively.

**Table 1:** Annotation of unigenes against seven public databases.

Databases	Number of Annotated unigenes	Annotation Ratio (%)
NR	67,065	61.00
NT	51,489	46.84
Swissprot	47,752	43.44
KEGG	53,451	48.62
COG	34,918	31.76
Interpro	49,617	45.13
GO	8,496	7.73
All annotated unigenes	72,287	65.75

### NR Annotation and COG Classification

**Figure 2:**
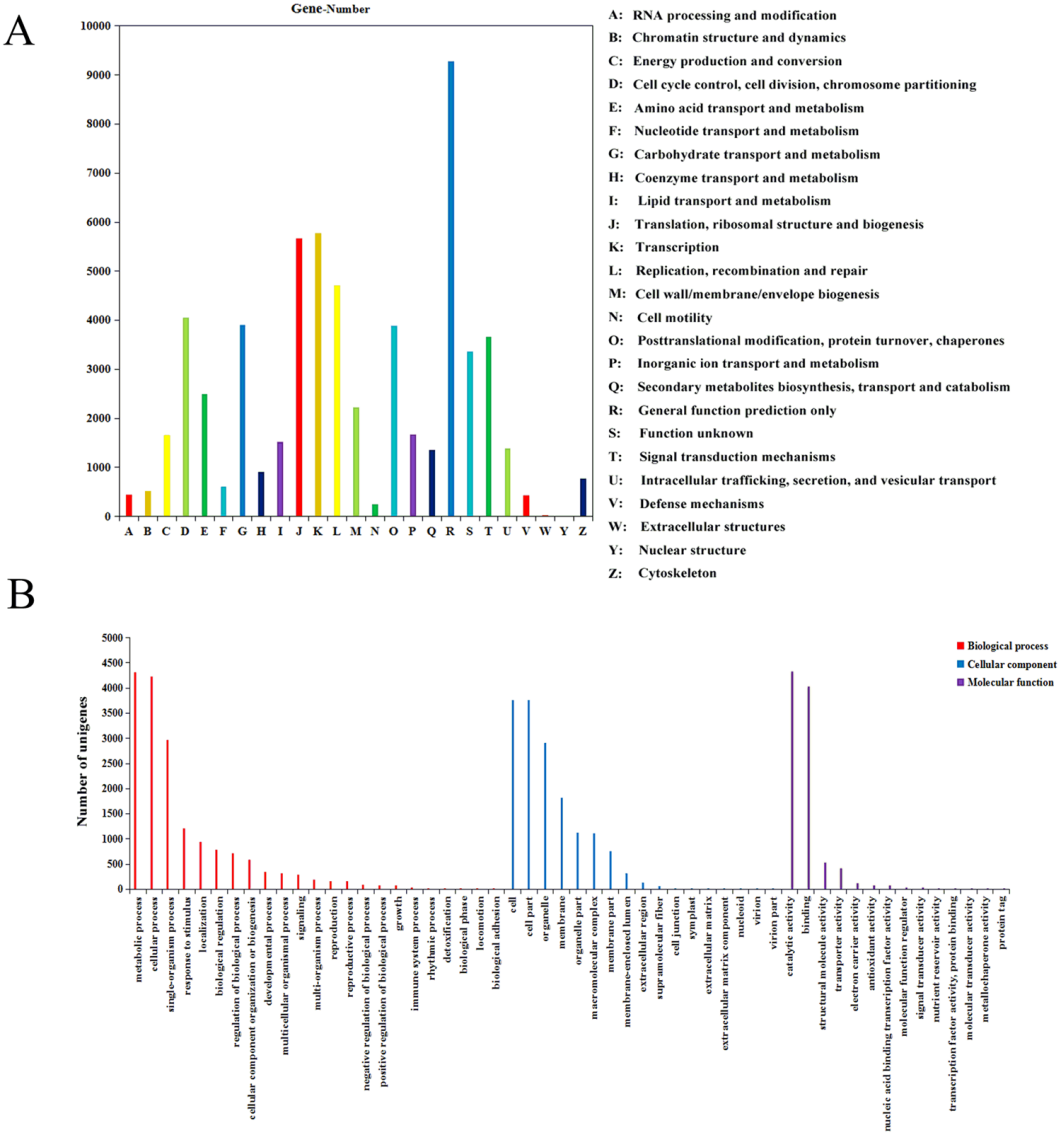
COG and GO annotation of *AhBl* transcriptome.

Nearly 61.00% of the assembled unigenes were aligned to NR protein database. Several species were searched by homologous unigenes, with 21.91% of the annotated unigenes have the highest similarity to unigene sequences from *Elaeis guineensis*, followed by *Phoenix dactylifera* (17.15%), and *Nelumbo nucifera* (9.36%) (Supplementary Fig. S3B). In order to further reveal the integrity of *AhBl* transcriptome, total 53,451 unigenes were annotated and assigned to COG classifications (Fig. 2A). Among the 25 COG classes, the cluster of “general function prediction only” (9,277, 26.57%) belonged to the largest proportion of the group, followed by “transcription” (5,784, 16.56%), and “translation, ribosomal structure and biogenesis” (5,667, 16.23%).

### GO Functional Classification

A total of 8,496 unigenes with GO annotation were allocated to GO classifications with three main categories (biological process, cellular component and molecular function) and 54 functional groups (Fig. 2B). Under the categories of biological process, two groups of prominently represented were scheduled for metabolic process (4,314 unigenes, 24.59%) and cellular process (4,236 unigenes, 24.14%). While the main terms in the cellular component were cell (3,758 unigenes, 23.70%) and cell part (3,758 unigenes, 23.70%). For the molecular function category, most of the unigenes were assigned to the catalytic activity (4,325 unigenes, 44.63%) and binding terms (4,031 unigenes, 41.60%).

### Identifcation of Candidate Genes Involved in Isoflavonoid Biosynthesis by KEGG Pathway Analysis

**Figure 3:**
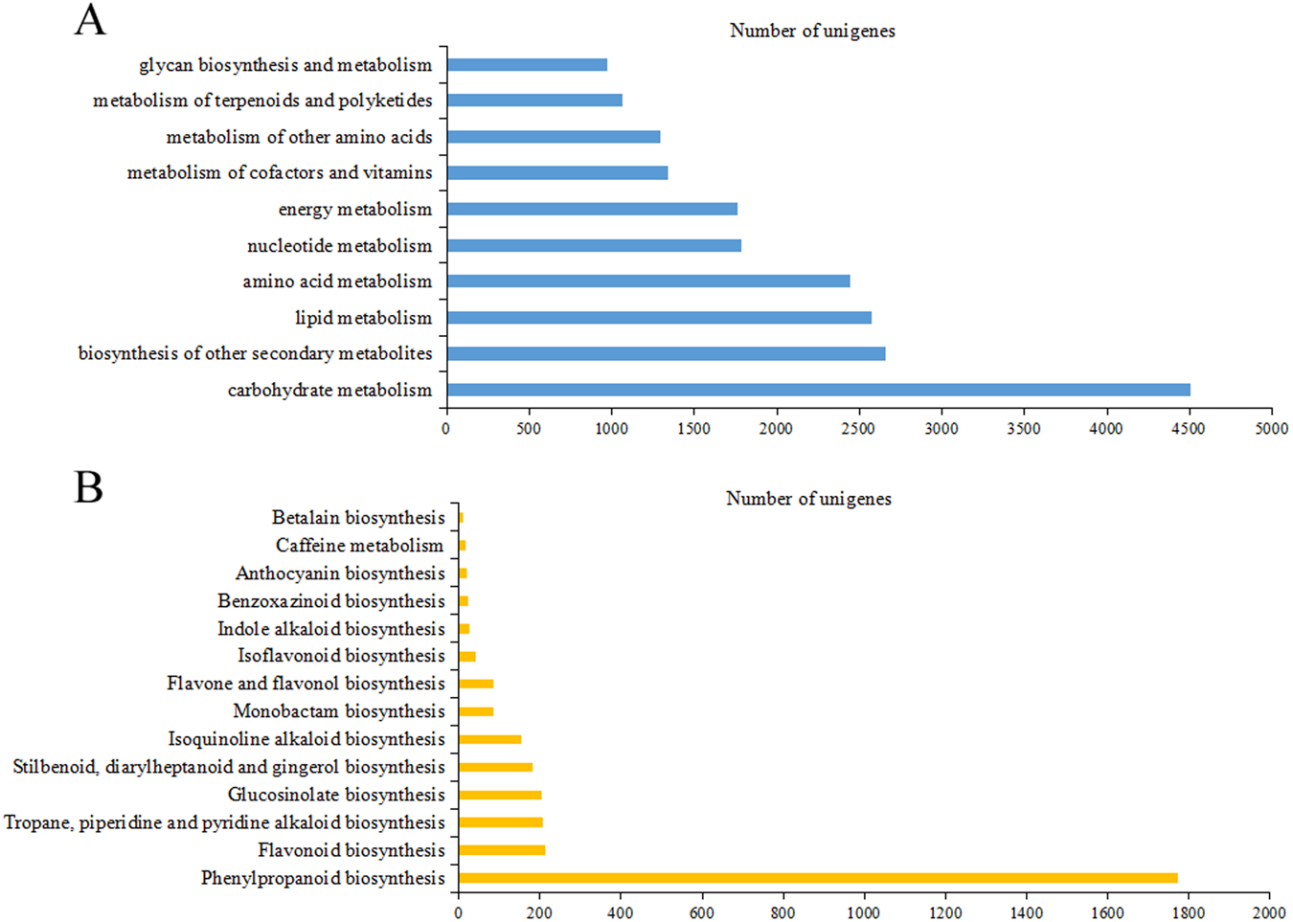
KEGG annotation of *AhBl* unigenes. (**A**) Classifications based on metabolism categories. (**B**) Classifications based on biosynthesis of other secondary metabolites.

To identify the main biological pathways, a total of 53,451 unigenes were mapped to canonical pathways and assigned to 137 pathways in KEGG database (Supplementary Table S4). The main categories of KEGG pathways included metabolism (33,182 unigenes), cellular processes (3,691 unigenes), environmental information processing (2,374 unigenes), genetic information processing (14,729 unigenes) and organismal systems (2,728 unigenes). In metabolism pathways, most of the genes were mainly distributed in carbohydrate metabolism (4,507 unigenes), followed by biosynthesis of other secondary metabolites (2,657 unigenes), lipid metabolism (2,578 unigenes), amino acid metabolism (2,443 unigenes), nucleotide metabolism (1,788 unigenes), energy metabolism (1,759 unigenes), metabolism of cofactors and vitamins (1,340 unigenes), metabolism of other amino acids (1,295 unigenes), metabolism of terpenoids and polyketides (1,066 unigenes), as well as glycan biosynthesis and metabolism (976 unigenes) (Fig. 3A).

**Figure 4:**
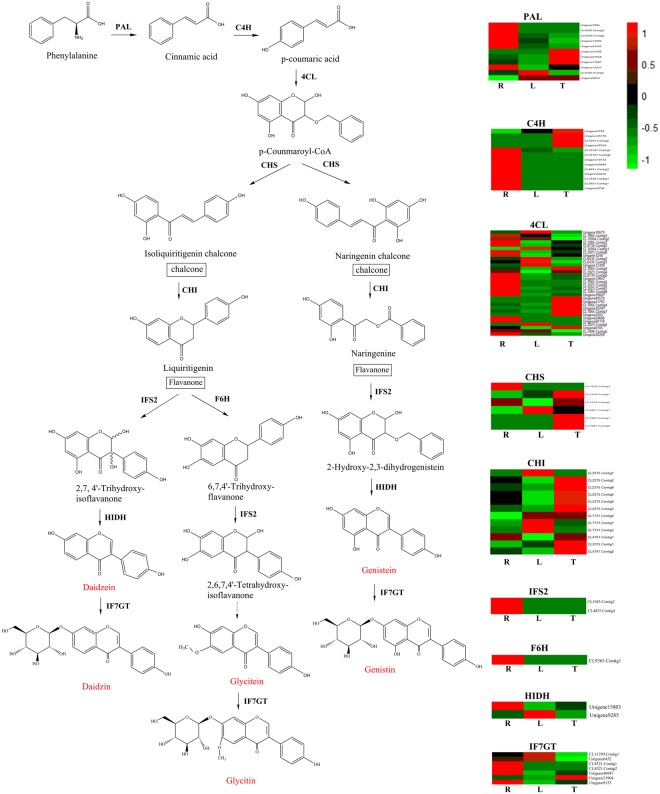
The isoflavonoid biosynthesis pathway in *AhBl*. The expression levels of the unigenes encoding enzymes for each process are shown using heatmap. The columns are R, L, T, corresponding to root, leaf and tuber, respectively, and the rows correspond to unigenes. Green and red represent low and high expression levels, respectively. Isoflavonoid products are marked in red.

The “biosynthesis of other secondary metabolites” subcategory contained 14 pathways, and the maximum number of unigenes was assigned to phenylpropanoid biosynthesis pathway (Fig. 3B). While the precursors for isoflavonoid biosnythesis were derived from the phenylpropanoid and flavonoid biosynthesis. A total of 87 unigenes encoding 9 key enzymes that regulate isoflavonoid biosynthesis were detected and differentially expressed genes encoding these enzymes were shown in Fig. 4, including phenylalanine ammonia lyase (PAL), 4-Coumarate-CoA ligase (4CL), trans-Cinnamate 4-monooxygenase (C4H), chalcone synthase (CHS), chalcone isomerase (CHI), 2-hydroxyisoflavanone synthase (IFS2), flavonoid 6-hydroxylase (F6H), 2-Hydroxyisoflavanone dehydratase (HIDH) and isoflavone 7-O- glucosyltransferase (IF7GT) (Table 2).

**Table 2:** Identification of unigenes involved in the isoflavonoid biosynthesis pathway.

Enzyme name	EC number	Unigene number	No. in roots	No. in tubers	No. in leaves
PAL	4.3.1.24	11	11	11	9
C4H	1.14.13.11	13	5	4	3
4CL	6.2.1.12	33	20	20	17
CHS	2.3.1.74	6	4	6	4
CHI	5.5.1.6	12	4	6	8
IFS2	1.14.13.136	2	1	1	1
F6H	1.14.13.-	1	0	0	0
HIDH	4.2.1.105	2	2	2	2
IF7GT	2.4.1.170	7	3	5	3

### Isoflavonoid Content Detection through High Performance Liquid Chromatography (HPLC)

The roots, tubers and leaves selected from three replicates were pooled together and assayed for isoflavonoid content. Total isoflavonoid content, including the five isoflavonoids, namely, daidzin, glycitin, genistin, daidzein and glycitein, was higher in tuber as compared to root or leaf by HPLC analysis (Supplementary Figs S4 and S5).

### Validation of Unigenes and Gene Expression Profiling Using qRT-PCR

**Figure 5:**
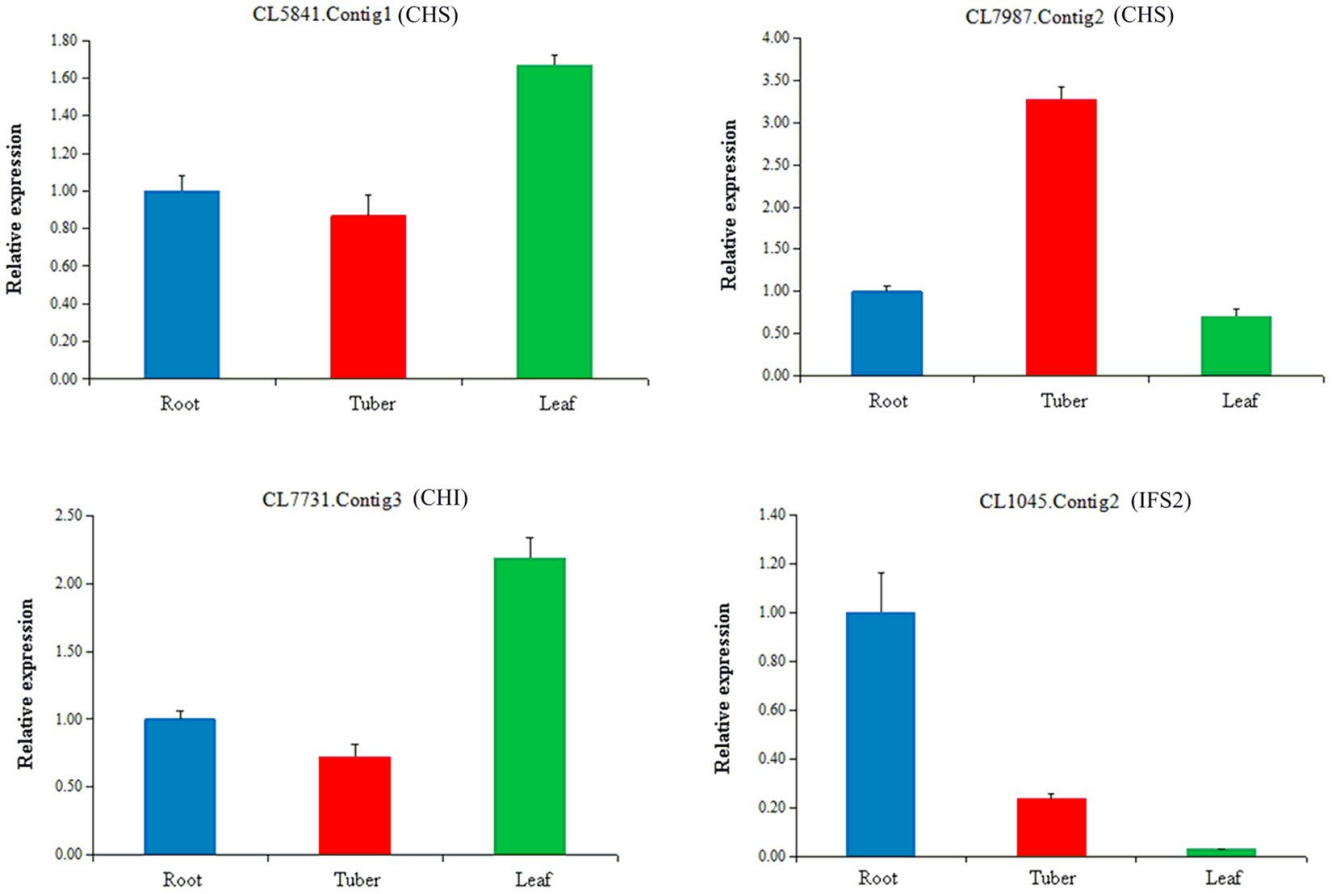
Real-Time PCR analysis of four unigenes involved in the isoflavonoid biosynthesis. Expression of the unigenes was analyzed, including CL5841.Contig1 (CHS), CL7987.Contig2 (CHS), CL7731.Contig3 (CHI) and CL1045.Contig2 (IFS2). Relative expression corresponds to average gene expression with technical triplicates. Error bars indicate SEM based on three replicates. Actin gene (CL4033.Contig1) was used as the reference genes for normalization.

To experimentally validate the expression profiles of the unigenes obtained from the assembled transcriptome, four significant differentially expressed unigenes involved in isoflavonoid biosynthesis, namely the unigenes CL5841.Contig1 and CL7987.Contig2 encoding CHS, CL7731.Contig3 encoding CHI, and CL1045.Contig2 encoding IFS2, were detected using qRT-PCR. As shown in Fig. 5, unigene CL7987.Contig2 in which tubers showed highest expression, unigene CL1045.Contig2 in which roots showed highest expression and the highest expression for CL5841.Contig1 and CL7731.Contig3 was in the leaves.

### Identification and Analysis of Differentially Expressed Genes (DEGs)

**Figure 6:**
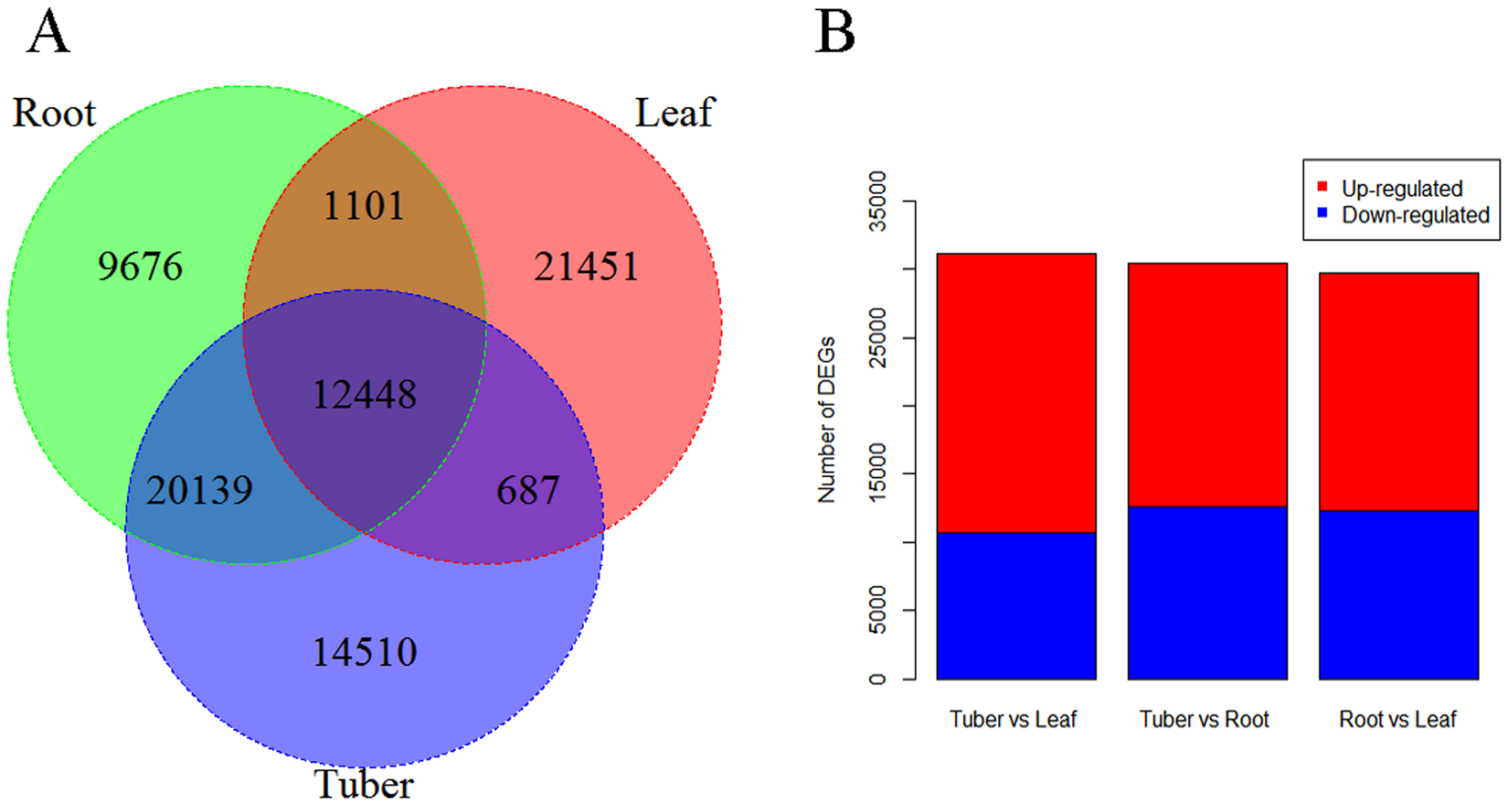
Unigenes expressed in different tissues of *AhBl*. (**A**) Venn diagram of unigenes expressed in different tissues of *AhBl*. (**B**) Differentially expressed unigene number among different tissues of *AhBl*. The numbers of up-regulated and down-regulated unigenes between root and leaf, root and tuber, and tuber and leaf are summarized. DEGs with higher expression levels in one tissue (such as root) when compared with another tissue (such as tuber) were denoted as up-regulated, while those with lower expression levels were denoted as down-regulated.

A venn diagram analysis of unigenes expressed in three different tissues of *AhBl* indicated that a total of 12,448 shared unigenes were identified and there were more unigenes expressed specifically in leaf than in root and tuber (Fig. 6A). To detect unigenes showing a significant differentially expressed among tissues, total 29,705 DEGs of the root and the leaf transcriptome were identified. Among them, 17,360 unigenes were regarded as up-regulated (higher expression in root) and 12,345 were regarded as down-regulated (lower expression in root). Between the tuber and leaf, 31,094 DEGs were checked with 20,430 up-regulated genes and 10,664 down-regulated genes. While 30,438 DEGs were checked with 17,853 up-regulated genes and 12,585 down-regulated genes between tuber and root (Fig. 6B).

**Figure 7:**
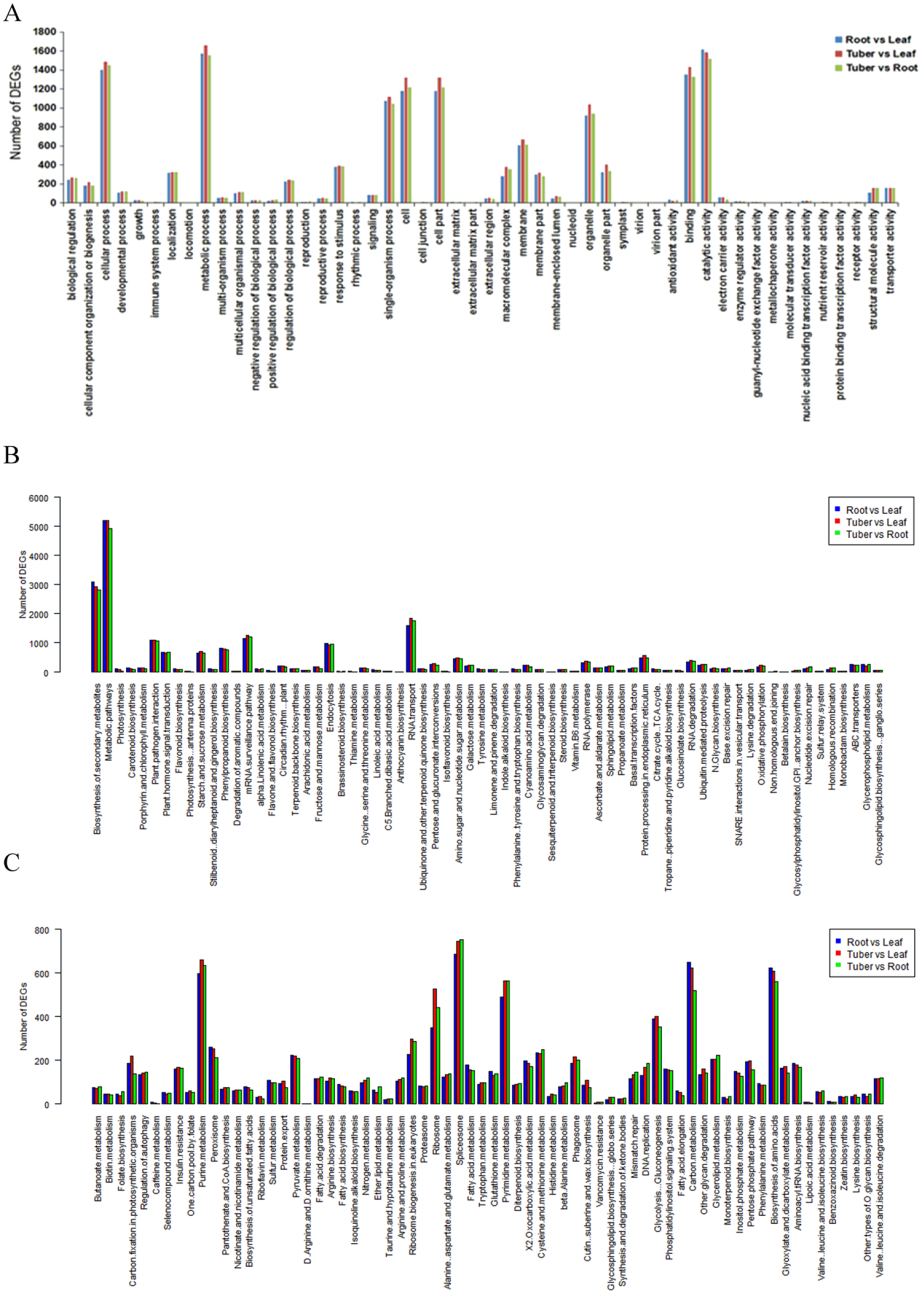
Analysis of DEGs annotated in GO terms and KEGG pathways. (**A**) GO classifications of DEGs. The categories of GO terms are represented on the X axis. Number of DEGs are represented on the Y axis. (**B**) and (**C**) KEGG annotation of DEGs among three different tissues of *AhBl*.

To test significantly enriched GO terms, all DEGs were mapped to GO databases. Among GO terms, molecular function, catalytic activity and binding were significantly enriched in DEGs between root vs. leaf and tuber vs. leaf (Fig. 7A). In biological process, most of the DEGs were clustered in metabolic process, cellular process and single-organism process. Mapping all DEGs to KEGG database, 136 pathways were specifically enriched in tuber vs. leaf (Fig. 7B, C), among which the most genes were enriched in metabolic pathways, biosynthesis of secondary metabolites and RNA transport. Other enriched pathways included phenylalanine, tyrosine and tryptophan biosynthesis, phenylpropanoid biosynthesis, flavonoid biosynthesis and isoflavonoid biosynthesis.

### Detection of Simple Sequence Repeats (SSRs)

**Figure 8:**
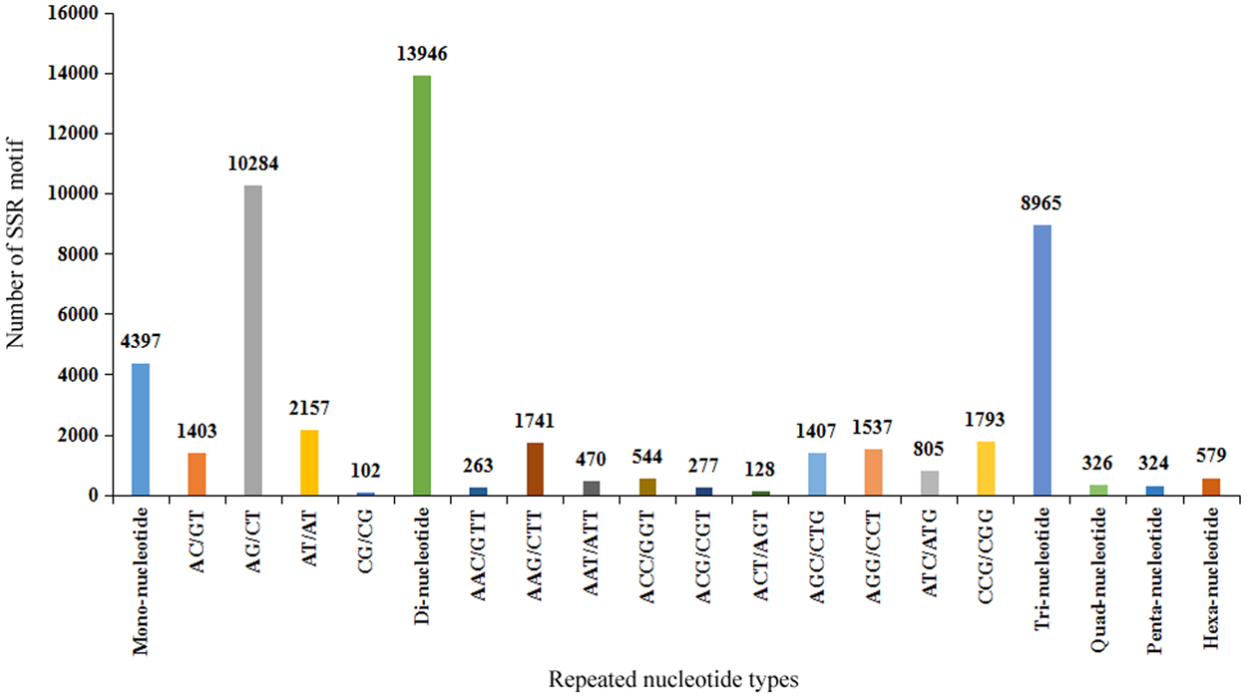
Types of SSR motifs in the *AhBl* transcriptome.

A total of 28,537 putative microsatellites were identified from 21,200 unigenes of the *AhBl* (Fig. 8). Among which seven types of SSRs were detected and 5,333 unigenes contained more than one SSR. There were 2,558 SSRs present in compound formation. Among 28,537 SSRs, the di-nucleotide repeat motifs (48.87%) were the highest proportion, followed by tri-nucleotide (31.42%), mono-nucleotide (15.41%), hexa-nucleotide (2.03%), quad-nucleotide (1.14%) and penta-nucleotide (1.14%).

## Discussion

The tuber of *AhBl* is considered to possess Pinellia-like medicinal herb properties. Despite the importance of its medicinal value, the genomic and transcriptomic data are unavailable. Here, we used Illumina HiSeq 4000 platform to establish the transcriptome of root, tuber and leaf of *AhBl* and performed de novo assembly and functional annotation to identify candidate unigenes involved in the isoflavonoid biosynthesis. The assembly results revealed that 109,937 unigenes were yielded, median length, N50 and GC content were 1,194 bp, 1,988 bp and 46.81%, respectively. Compared with previous studies, the median length and N50 sizes of unigenes in this study were longer than those assembled in *Pinellia ternata*^24^ (median length = 750 bp, N50 = 1,112 bp), *Platycodon grandiflorum*^25^ (median length = 1,102 bp, N50 = 1,796 bp), *rubber tree*^26^ (median length = 485 bp, N50 = 592 bp), and *Camellia sinensis*^27^ (median length = 355 bp, N50 = 506 bp). These results indicated that the transcriptome data from *AhBl* were effectively assembled. Nevertheless, only about 43% clean reads were de novo assembled into unigenes, which is lesser than other studies^28,29^, suggesting that there was much information unavailable in the transcriptome of *AhBl*.

In comparison with root and leaf, there was more up-regulated transcripts in tuber (Fig. 6B). According to the DEGs analysis results (Fig. 7A,B), the number of unigenes involved in metabolic pathways in tuber were more than in leaf and root. Meanwhile, 10,215 tuber-specific expressed unigenes were counted based on the FPKM values in three tissues. Moreover, 14,510 unigenes were uniquely expressed in tuber. These results may support the tuber of *AhBl* as a Chinese medicinal material at the genetic level.

Transcriptome is an important resource for the development of genetic diversity analysis, comparative genomics, and potential molecular marker-assisted selection in plant breeding^30,31^. Here, we identified 28,537 SSRs in 21,200 unigenes. Although the screening criteria for EST-SSR markers development in this study were different, the major types of the EST-SSR markers were di-nucleotide and tri-nucleotide, which linked to previous studies^32,33^. The largest fraction of di-nucleotide and tri-nucleotide motifs were AG/TC (73.39%) and CCG/CGG (20.00%), respectively (Fig. 8). And the AG/TC was the most abundant motif of di-nucleotide, which was consistent with prior reports^32,33^. The 28,537 SSRs identified in this transcriptome will provide a valuable resource to develop EST-SSRs in *AhBl*.

In this study, numerous unigenes involved in isoflavonoid biosynthesis were identifed on the basis of KEGG database. In addition, the expression levels of unigenes encoding enzymes in the phenylpropanoid and flavonoids pathways were analyzed based on FPKM values (Fig. 4). The unigenes encoding PAL, C4H, 4CL, IFS2, F6H and IF7GT were higher expression in the roots, while the unigenes encoding CHS and CHI showed higher expression in the tubers. It’s reported that CHS and CHI were the key enzymes in isoflavonoid synthesis, and the levels of these genes expression directly affected the content of isoflavonoid^34–36^. The expression levels of the unigenes encoding CHS, CHI, and IFS2 were experimentally validated by qRT-PCR, which confirmed the reliability of our transcriptional data (Figs 4 and 5). Furthermore, the high-level expression of CL7987.Contig2 gene (encoding CHS) in the tubers as analyzed by qRT-PCR was consistent with the isoflavonoid accumulation profiles in the tubers of *AhBl* via HPLC (Supplementary Figs S4 and S5), which suggested that the gene may play a vital role in the synthesis of the isoflavonoid.

In summary, using de novo transcriptome assembly, we assembled and annotated 109,937 and 72,287 unigenes from roots, tubers and leaves tissues of *AhBl*, respectively. We found the unigenes encoding key enzymes involved in the biosynthesis of isoflavonoid. Further study on the regulation of the expression of these key enzyme genes may greatly improve the essential production of isoflavonoid. Our transcriptomic dataset will be valuable for improving further research on *AhBl* functional genomics.

## Materials and Methods

### Plant Material and RNA Extraction

Whole *AhBl* plants were picked from the medicine garden, Anhui University of Chinese Medicine. The tissues (roots, tubers and leaves) of this plant were separated and immediately placed in liquid nitrogen refrigeration to freeze, storing at −80 °C to avoid RNA degradation. The roots, tubers and leaves selected from three independent biological replicates were pooled together. Total RNA from each tissue was used for cDNA preparation with E.Z.N.A Plant RNA Kit (50) (OMEGA Bio-Tek, USA) following the manufacturer’s instructions. RNA concentration, 28S/18S and RNA integrity number (RIN) were checked using the Agilent 2100. NanoDrop was used for the detection of the OD260/280 and OD260/230 ratios (Supplementary Table S5).

### cDNA Library Construction and Sequencing

The mRNA was enriched from total RNA using Oligo (dT) beads according to the manufacturer’s instructions. After purification, the mRNA was immediately fragmented in the Illumina fragmentation buffer and reverse transcription to synthesize first strand cDNA with the mRNA fragmentsas templates. Second-strand cDNA synthesis was conducted using dNTPs, RNase H and DNA polymerase I. Short cDNA fragments were further processed through end repair and ligation of adaptors with Illumina paired-end adapter oligo nucleotides. After that, to preferentially select the appropriate cDNA fragments, the products were purified and used for PCR amplification. The quantification of each cDNA library was detected via Agilent 2100 Bioanalyzer and ABI StepOnePlus Real-Time PCR System. Then the cDNA libraries were constructed using Illumina HiSeq 4000 technology and 32.93 Gb paired-end reads were generated.

### Transcriptome De novo Assembly

Before assembly, the raw reads with low quality (above 50% of bases with Q-value ≤ 20), ambiguous reads, adaptor sequences and duplication sequences were removed. A process of transcriptome assembly was described previously^37^. The transcriptome assembler, Trinity^38^, was performed by default parameters (K-mer = 25, group pairs distance = 400) with the following command: Trinity.pl–seqTypefq–left reads_1.fq–right reads_2.fq–max_memory 50 G–CPU 8. The assembled transcripts were extended and clustered using the TGICL software^39^. All transcripts were conducted on Illumina HiSeq 4000 platform. The assembled transcripts were processed for further functional annotation and classification analysis.

### Gene Expression Analysis and Functional Classification

To estimate the overall gene expression, quantitative method was adopted to calculate the number of Illumina reads using Bowtie2 with default parameters^40^, which represented each unigene expression level of each tissue. Then the numbers of expressed unigenes were calculated based on fragments per kilobase of transcript per million mapped reads (FPKM > 1)^41^ by RSEM (RNA-Seq by Expectation-Maximization) software to standardize the expression of genes^42^.

For acquiring unigenes functional annotation, all-unigenes were aligned against protein databases in NCBI such as NR (http://www.ncbi.nlm.nih.gov/), NT (ftp://ftp.ncbi.nlm.nil.gov/blast/db), SwissProt (http://www.expasy.ch/sport), COG (http://www.ncbi.nlm.nih.gov/cog), KEGG (http://www.genome.jp/kegg) with BLASTx program (E-value ≤ 1e-5). Blast2GO program^43^ was run to get GO annotation of every unigene. Afterwards, we obtained GO functional classifications for all unigenes using WEGO software^44^ to understand the distribution of gene functions. InterPro annotations (http://www.ebi.ac.uk/interpro) were got based on InterProScan5 program. Furthermore, the unigenes were also mapped back to COG database for predicting and analyzing possible functional categories. Pathway distributions were performed based on KEGG pathway database^45^.

### Analysis of Differentially Expressed Genes

Differentially expressed genes (DEGs) were identified by PossionDis methods^46^ based on the poisson distribution. To screen DEGs, p values corresponding to DEGs were performed as described at Audic S, *et al.*^47^. The thresholds of p values were corrected in multiple hypothesis tests via the modulation of FDR (false discovery rate) value. Ultimately, the unigenes with ratios of FC (fold change) ≥ 2.00 and FDR ≤ 0.001 were defined as significant differences in expression.

In GO functional analysis, a hypergeometric test was used for all DEGs mapped to terms in GO database, so as to detect significantly enriched GO terms in DEGs compared with the whole transcriptome of *AhBl*. The p value method was as follows:

$$p=1-\sum _{i=0}^{m-1}(M{\rm{i}})(N-Mn-i)/(Nn)$$

where N, n, M and m were the number of annotated unigenes with GO annotations, DEGs in N, annotated unigenes corresponded to the certain GO term and DEGs in M, respectively. KEGG, a database related to the pathway, was used as signal transduction or significantly enriched metabolic pathways for identification compared to the transcriptome background. The p value method was described as the previous GO annotations analysis. The main signal transduction pathways and metabolic pathways involved in DEGs were identified.

### Identification of SSRs

SSRs markers were identified in the 21,200 unigenes of *AhBl* using the MISA (Micro SAtellite) Tool^48^. Based on screened by MISA, we obtained Mono-, Di-, Tri-, Quad-, Penta- and Hexa-nucleotide motifs with the set parameters of 1/12, 2/6, 3/5, 4/5, 5/4 and 6/4 (unitsize/minimize repeats). A maximum distance was defined as 100 base pairs between two SSRs.

### Isoflavonoid Content Detection by HPLC

Isoflavonoid was detected by HPLC under the following conditions: C18 chromatographic column (JADE-PAK ODS-AQ) (250 mm × 4.6 mm, 5.0 μm); mobile phase: acetonitrile- phosphoric acid (30:70, v/v); flow rate 1.0 ml/min; wavelength = 260 nm; column temperature 40 °C. All detections were performed in triplicate for each sample.

### qRT-PCR Analysis of Key Genes in Isoflavonoid Biosynthesis

CHS, CHI and IFS genes potentially involved in isoflavonoid biosynthesis were selected for qRT-PCR experiments. qRT-PCR was performed using QuantiNova SyBr Green PCR kit (Qiagen) on CFX96™ RealTime Detection System (Bio-Rad, USA). Unigene-specific primers for qRT-PCR were designed using the Primer v5.0 software (Supplementary Table S6). Total volume of the reaction system was 10 μL, including 2.0 μL cDNA, 5 uL template SYBR Green mixture (2×), 1.0 μL of forward and reverse primer and 2.0 μL of RNase free water.

The amplification condition was as follows: 95 °C for 2 min, followed by 40 cycles of 95 °C for 5 s, 60 °C for 10 s. The relative abundance of each unigene was expressed as mean ± standard deviation (SD) and the relative expression levels of selected unigenes were normalized to actin gene (CL4033.Contig1) and evaluated using the 2^−ΔΔCt^ method^49^. All reactions were performed in triplicate for each sample. Melting curves were generated for each sample to determine amplification specificity.

## Electronic supplementary material

supplementary material

## Data Availability

The RNA-seq datasets of three *AhBl* tissues have been deposited in NCBI Sequence Read Archive (SRA) database (Accession: SRP118752).
