# Supplementary material for: De novo sequencing and transcriptome assembly of Arisaema heterophyllum Blume and identification of genes involved in isoflavonoid biosynthesis
Source: Sci Rep. 2018 Dec 5;8:17643. doi: 10.1038/s41598-018-35664-1 (PMC6281570; doi:10.1038/s41598-018-35664-1)
Supplement: Supplementary file 1 — supplementary material [file 41598_2018_35664_MOESM1_ESM.pdf]

De novo sequencing and transcriptome assembly of *Arisaema heterophyllum* Blume and identification of genes involved in isoflavonoid biosynthesis

Chenkai Wang[1,3†], Jinhang Zhu[2†], Miaomiao Liu[1,3], Qingshan Yang[1,4], Jiawen Wu[1,3,4*], Zegeng Li[1,5,6*].

[1]Anhui University of Chinese Medicine and Anhui Academy of Chinese Medicine, Hefei 230038, China.

[2]Anhui Medical University, Hefei 230032, China.

[3]Key Laboratory of Xin'an Medicine, Ministry of Education, Anhui University of Chinese Medicine, Hefei 230038, China.

[4]Synergetic Innovation Center of Anhui Authentic Chinese Medicine Quality Improvement, Hefei 230012, China.

[5]The First Affiliated Hospital of Anhui University of traditional Chinese Medicine, Anhui 230038, China.

[6]Key Laboratory of Respiratory Diseases, State Administration of Traditional Chinese Medicine of the People's Republic of China, Anhui 230038, China.

[†]Chenkai Wang and Jinhang Zhu contributed equally to this work.

*Corresponding author. Jiawen Wu, Zegeng Li, E-mail address: wujiawen@ahtcm.edu.cn, li6609@126.com

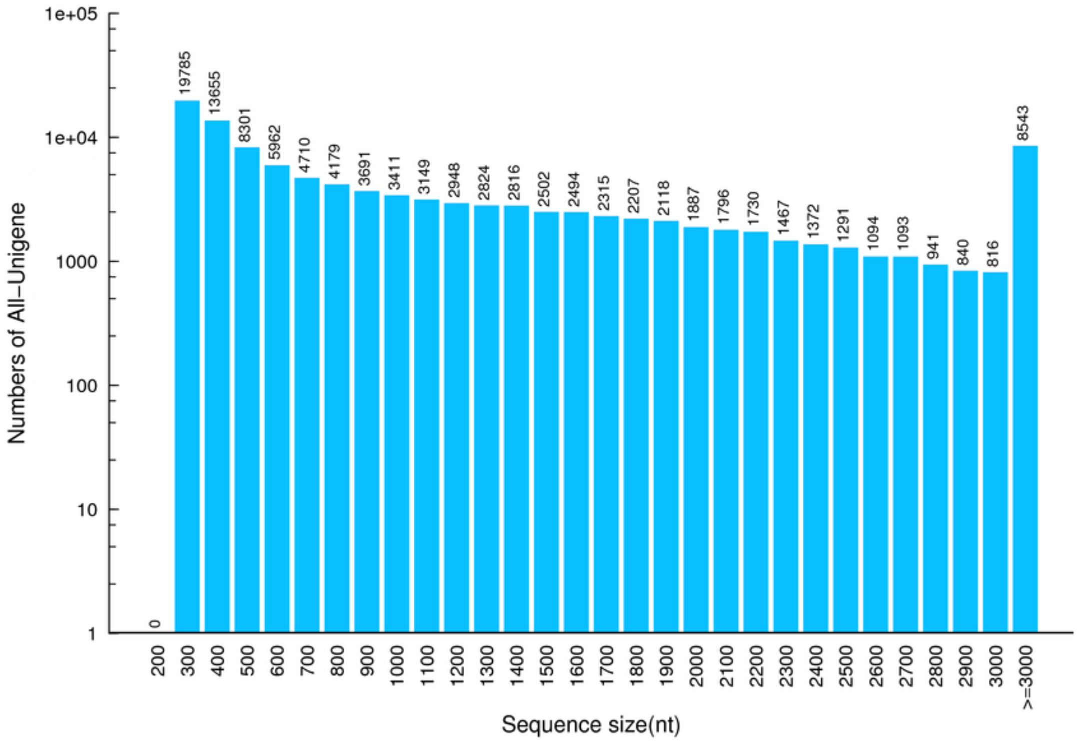

Supplementary Figure S1. Distribution of unigene sizes from *AhBl*.

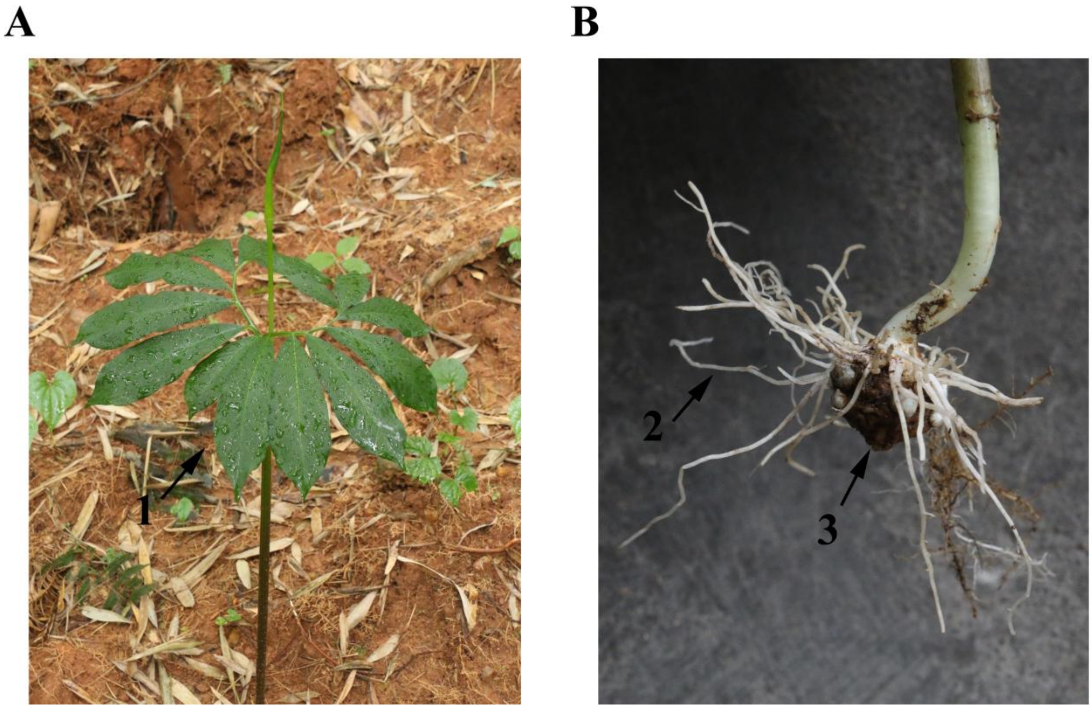

Supplementary Figure S2. Pictures of *AhBl* plant. 1, 2 and 3 represent leaf, root and tuber, respectively.

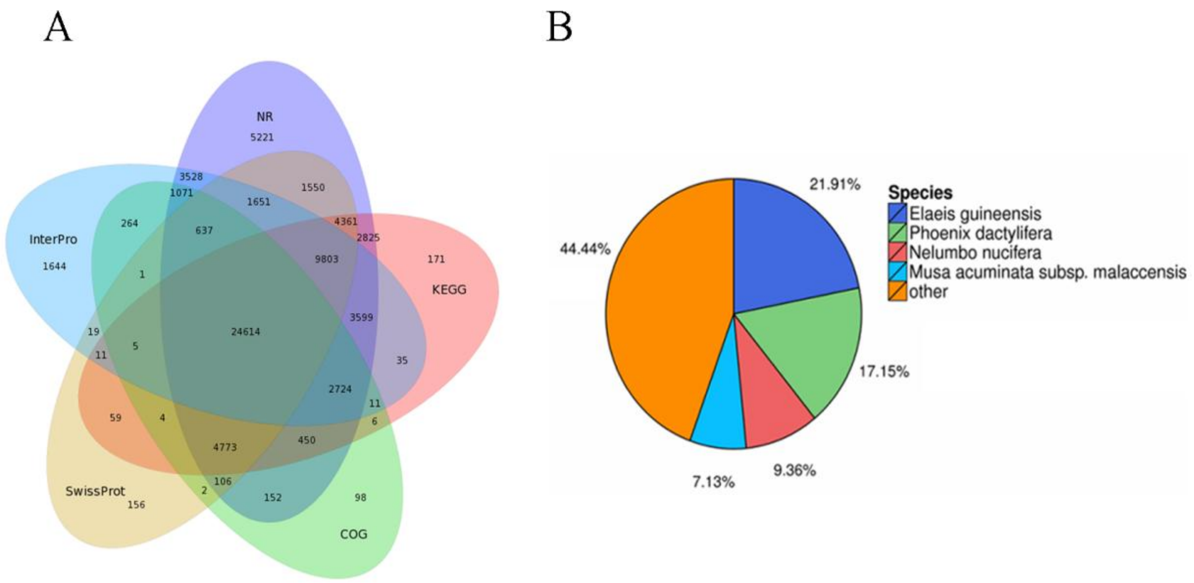

Supplementary Figure S3. (A) Venn diagram of annotated unigenes from the different databases. (B) Homologous species distribution annotated in the NR database for *AhBl*.

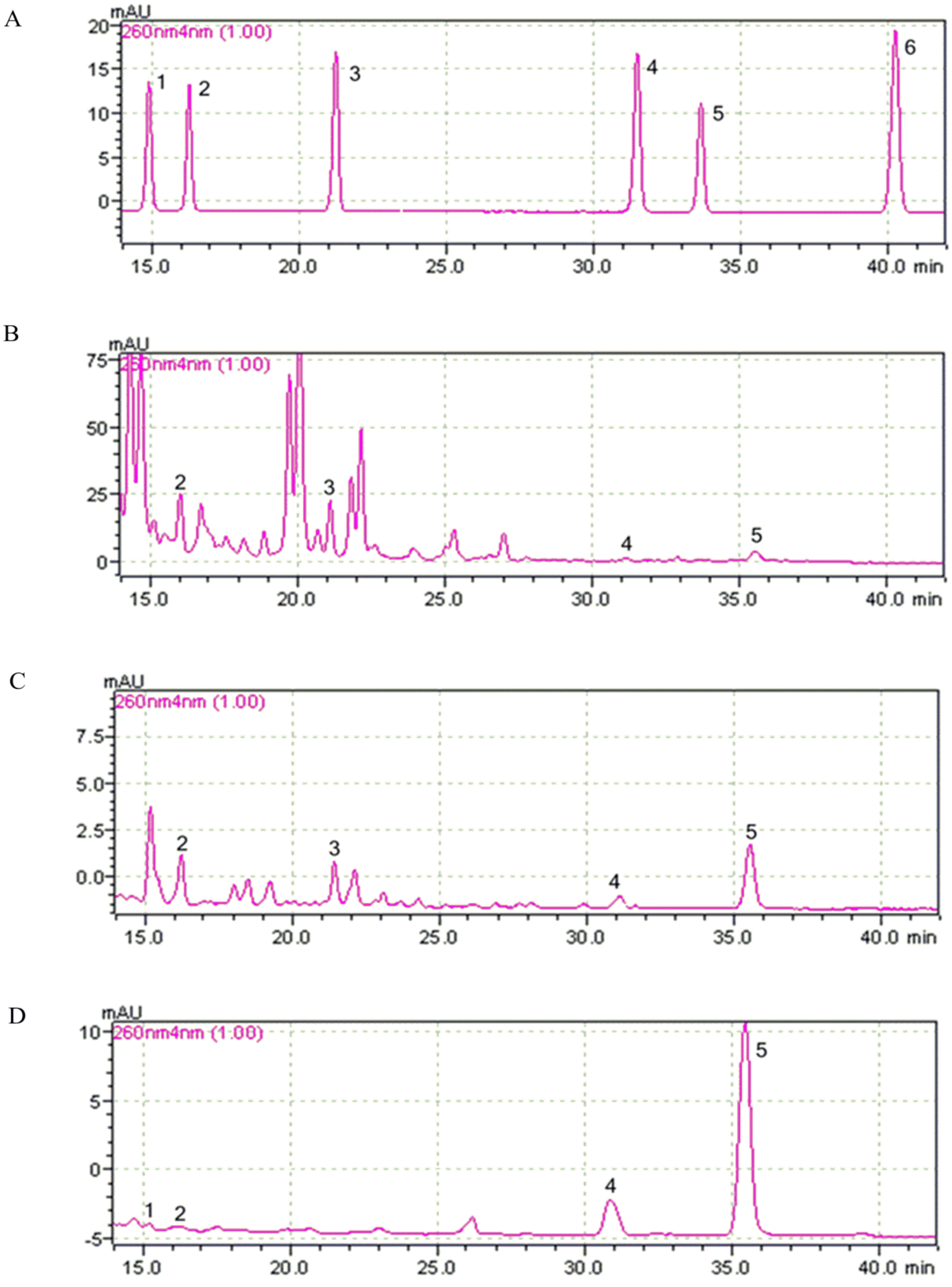

Supplementary Figure S4. The content of isoflavonoid was detected by HPLC in different *AhBl* tissues. A is the HPLC map of reference solution. B, C and D are the HPLC maps of test sample (leaf, root and tuber) solutions. 1, 2, 3, 4, 5 and 6 represent daidzin, glycitin, genistin, daidzein, glycitein and genistein, respectively.

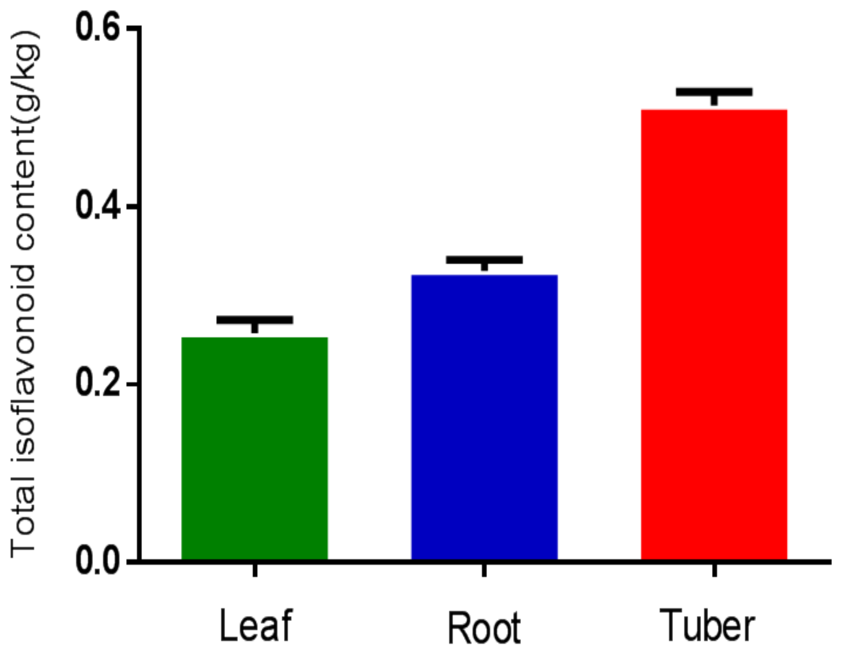

Supplementary Figure S5. Total isoflavonoid content of three *AhBl* tissues. Total isoflavonoid content, including the five isoflavonoids, namely, daidzein, glycitein, daidzin, genistin and glycitin, was measured by HPLC. Error bars indicated SEM of three independent experiments.

Supplementary Table S1. Summary of sequencing reads after filtering.

Sample	Total Raw Reads (Mb)	Total Clean Reads (Mb)	Total Clean Bases (Gb)	Clean Reads Q20 (%)	Clean Reads Q30 (%)	Clean Reads Ratio (%)
Leaf	83.17	73.53	11.03	95.72	89.06	88.42
Root	83.63	73.63	11.04	95.73	89.08	88.05
Tuber	81.75	72.37	10.86	96.22	90.12	88.53

Supplementary Table S2. Quality metrics of transcripts.

Sample	Total Number	Total Length	Mean Length	N50	N70	GC (%)
Leaf	64,434	72,598,686	1,126	1,803	1,192	47.95
Root	89,452	84,460,343	944	1,592	919	47
Tuber	101,600	107,388,793	1,056	1,843	1,110	47.11

Supplementary Table S3. Qualitative unigene index.

Sample	Total Number	Total Length	Mean Length	N50	N70	GC (%)
Leaf	51,310	64,154,515	1,250	1,906	1,321	47.92
Root	67,957	72,489,364	1,066	1,744	1,095	46.84
Tuber	80,957	95,079,096	1,174	1,985	1,280	47.09
All-Unigene	109,937	131,342,529	1,194	1,988	1,296	46.81

Supplementary Table S4. KEGG annotation of all unigenes.

Pathway	Unigene Number	Pathway ID
Metabolic pathways	12047	ko01100
Biosynthesis of secondary metabolites	6876	ko01110
RNA transport	3813	ko03013
mRNA surveillance pathway	2590	ko03015
Plant-pathogen interaction	2348	ko04626
Endocytosis	2250	ko04144
Spliceosome	2103	ko03040
Phenylpropanoid biosynthesis	1771	ko00940
Carbon metabolism	1582	ko01200
Biosynthesis of amino acids	1534	ko01230
Protein processing in endoplasmic reticulum	1503	ko04141
Purine metabolism	1489	ko00230
Ribosome	1479	ko03010
Plant hormone signal transduction	1404	ko04075
Starch and sucrose metabolism	1379	ko00500
Pyrimidine metabolism	1275	ko00240
Amino sugar and nucleotide sugar metabolism	1058	ko00520
RNA degradation	947	ko03018
Glycolysis / Gluconeogenesis	926	ko00010
Ribosome biogenesis in eukaryotes	847	ko03008
RNA polymerase	747	ko03020
Ubiquitin mediated proteolysis	698	ko04120
Peroxisome	648	ko04146
Cysteine and methionine metabolism	635	ko00270
ABC transporters	609	ko02010
Oxidative phosphorylation	599	ko00190
Glycerophospholipid metabolism	591	ko00564
Pentose and glucuronate interconversions	569	ko00040
Pyruvate metabolism	566	ko00620
Cyanoamino acid metabolism	540	ko00460
2-Oxocarboxylic acid metabolism	533	ko01210
DNA replication	531	ko03030
Phagosome	525	ko04145
Nucleotide excision repair	507	ko03420
Glycerolipid metabolism	482	ko00561
Galactose metabolism	475	ko00052
Fatty acid metabolism	466	ko01212
Carbon fixation in photosynthetic organisms	460	ko00710
Pentose phosphate pathway	456	ko00030
Aminoacyl-tRNA biosynthesis	444	ko00970

Mismatch repair	420	ko03430
Homologous recombination	409	ko03440
Insulin resistance	399	ko04931
Glutathione metabolism	396	ko00480
Sphingolipid metabolism	393	ko00600
Glyoxylate and dicarboxylate metabolism	390	ko00630
Circadian rhythm - plant	384	ko04712
Phosphatidylinositol signaling system	370	ko04070
Fructose and mannose metabolism	369	ko00051
Basal transcription factors	360	ko03022
Inositol phosphate metabolism	351	ko00562
Ascorbate and aldarate metabolism	345	ko00053
Valine, leucine and isoleucine degradation	337	ko00280
N-Glycan biosynthesis	336	ko00510
Base excision repair	336	ko03410
Regulation of autophagy	332	ko04140
Glycine, serine and threonine metabolism	331	ko00260
Alanine, aspartate and glutamate metabolism	331	ko00250
Other glycan degradation	321	ko00511
Citrate cycle (TCA cycle)	319	ko00020
Fatty acid degradation	296	ko00071
Arginine and proline metabolism	277	ko00330
Sulfur metabolism	274	ko00920
Arginine biosynthesis	272	ko00220
Cutin, suberine and wax biosynthesis	265	ko00073
Lysine degradation	262	ko00310
Nitrogen metabolism	258	ko00910
Porphyrin and chlorophyll metabolism	249	ko00860
Phenylalanine, tyrosine and tryptophan biosynthesis	249	ko00400
Tryptophan metabolism	246	ko00380
Tyrosine metabolism	246	ko00350
Terpenoid backbone biosynthesis	246	ko00900
Ubiquinone and other terpenoid-quinone biosynthesis	246	ko00130
Diterpenoid biosynthesis	243	ko00904
Protein export	239	ko03060
Fatty acid biosynthesis	234	ko00061
beta-Alanine metabolism	228	ko00410
alpha-Linolenic acid metabolism	228	ko00592
Proteasome	225	ko03050
Phenylalanine metabolism	221	ko00360
Flavonoid biosynthesis	214	ko00941

Carotenoid biosynthesis	207	ko00906
Tropane, piperidine and pyridine alkaloid biosynthesis	206	ko00960
Glucosinolate biosynthesis	204	ko00966
Biosynthesis of unsaturated fatty acids	199	ko01040
Steroid biosynthesis	199	ko00100
Propanoate metabolism	192	ko00640
Limonene and pinene degradation	182	ko00903
Stilbenoid, diarylheptanoid and gingerol biosynthesis	182	ko00945
Butanoate metabolism	182	ko00650
Glycosaminoglycan degradation	180	ko00531
SNARE interactions in vesicular transport	176	ko04130
Pantothenate and CoA biosynthesis	171	ko00770
Ether lipid metabolism	171	ko00565
Linoleic acid metabolism	157	ko00591
Nicotinate and nicotinamide metabolism	156	ko00760
Isoquinoline alkaloid biosynthesis	155	ko00950
Photosynthesis	151	ko00195
Glycosylphosphatidylinositol(GPI)-anchor biosynthesis	148	ko00563
Glycosphingolipid biosynthesis - ganglio series	143	ko00604
Fatty acid elongation	138	ko00062
Selenocompound metabolism	133	ko00450
Valine, leucine and isoleucine biosynthesis	133	ko00290
One carbon pool by folate	132	ko00670
Arachidonic acid metabolism	127	ko00590
Folate biosynthesis	113	ko00790
Biotin metabolism	104	ko00780
Histidine metabolism	102	ko00340
Sulfur relay system	98	ko04122
Zeatin biosynthesis	97	ko00908
Monobactam biosynthesis	85	ko00261
Flavone and flavonol biosynthesis	84	ko00944
Other types of O-glycan biosynthesis	80	ko00514
Lysine biosynthesis	76	ko00300
Riboflavin metabolism	76	ko00740
C5-Branched dibasic acid metabolism	70	ko00660
Monoterpenoid biosynthesis	70	ko00902
Synthesis and degradation of ketone bodies	68	ko00072
Degradation of aromatic compounds	67	ko01220
Vitamin B6 metabolism	64	ko00750
Brassinosteroid biosynthesis	61	ko00905

Glycosphingolipid biosynthesis - globo series	57	ko00603
Taurine and hypotaurine metabolism	53	ko00430
Non-homologous end-joining	53	ko03450
Isoflavonoid biosynthesis	42	ko00943
Sesquiterpenoid and triterpenoid biosynthesis	39	ko00909
Thiamine metabolism	39	ko00730
Photosynthesis - antenna proteins	34	ko00196
Indole alkaloid biosynthesis	25	ko00901
Benzoxazinoid biosynthesis	24	ko00402
Anthocyanin biosynthesis	21	ko00942
Lipoic acid metabolism	16	ko00785
Caffeine metabolism	16	ko00232
Vancomycin resistance	13	ko01502
Betalain biosynthesis	11	ko00965
D-Arginine and D-ornithine metabolism	2	ko00472
Carbapenem biosynthesis	1	ko00332

Supplementary Table S5. RNA information of different tissues.

Sample	Concentration (ng / μL)	Total amount (μg)	OD260/280	RIN	28S/18S
Leaves	201	6.03	2.09	6.9	1.1
Roots	101	2.02	2.03	9.3	1.3
Tubers	210	10.5	1.61	8.7	1.3

Supplementary Table S6. Target genes and primers for qRT-PCR.

Genes	Primer pairs	length(bp)	Tm value
CL5841.Contig1	5'-ACATCACCCACCTCGTCTAC-3'	20	56.6
	5'-CAGCCGAGGAAGTAGAGCAT-3'	20	57
CL7987.Contig2	5'-CGAGGCGAAACTGAACTTGA-3'	20	55.3
	5'-AATAACCGTCGGACTCGACA-3'	20	55.8
CL7731.Contig3	5'-CGCCCTCTTAAGCAAGGAAC-3'	20	56.4
	5'-CGAACAAGGCAAAGGTGACA-3'	20	55
CL1045.Contig2	5'-CCAATCCACTTTACTGGAAAG-3'	21	50.7
	5'-TCAATCATTGCAATCTTTCT-3'	20	46
Actin	5'-GACGTCGTGTGGAATCACTG-3'	20	56.2
	5'-TGAATTACCACGTCCGTCCA-3'	20	56.1
